# Cross-linguistic patterns of speech prosodic differences in autism: A machine learning study

**DOI:** 10.1371/journal.pone.0269637

**Published:** 2022-06-08

**Authors:** Joseph C. Y. Lau, Shivani Patel, Xin Kang, Kritika Nayar, Gary E. Martin, Jason Choy, Patrick C. M. Wong, Molly Losh

**Affiliations:** 1 Roxelyn and Richard Pepper Department of Communication Sciences and Disorders, Northwestern University, Evanston, Illinois, United States of America; 2 Department of Linguistics and Modern Languages, The Chinese University of Hong Kong, Hong Kong S.A.R., China; 3 Brain and Mind Institute, The Chinese University of Hong Kong, Hong Kong S.A.R., China; 4 Research Centre for Language, Cognition and Language Application, Chongqing University, Chongqing, China; 5 School of Foreign Languages and Cultures, Chongqing University, Chongqing, China; 6 Department of Communication Sciences and Disorders, St. John’s University, Staten Island, New York, United States of America; PLOS, UNITED KINGDOM

## Abstract

Differences in speech prosody are a widely observed feature of Autism Spectrum Disorder (ASD). However, it is unclear how prosodic differences in ASD manifest across different languages that demonstrate cross-linguistic variability in prosody. Using a supervised machine-learning analytic approach, we examined acoustic features relevant to rhythmic and intonational aspects of prosody derived from narrative samples elicited in English and Cantonese, two typologically and prosodically distinct languages. Our models revealed successful classification of ASD diagnosis using rhythm-relative features within and across both languages. Classification with intonation-relevant features was significant for English but not Cantonese. Results highlight differences in rhythm as a key prosodic feature impacted in ASD, and also demonstrate important variability in other prosodic properties that appear to be modulated by language-specific differences, such as intonation.

## Introduction

Differences in prosody are observed in many individuals with autism spectrum disorder (ASD) [1, 2]. Although they may manifest variably across individuals and across different language contexts (e.g., [3–5], also see [2, 6] for reviews), prosodic differences have been considered a central feature of communication profile of ASD [7–10]. Speech prosody involves the use of rhythm (i.e., variation of regularity in loudness and speed) and intonation (i.e., variation of voice pitch) [6] to encode grammatical information, represent pragmatic information, as well as to express speaker intent and emotion [11–13]. Differences in prosody can significantly undermine social and communicative competence by disrupting communication of this linguistic and meta-linguistic information and contrasts (e.g., parts of speech differences [e.g., CONtent vs. conTENT]; a sarcastic vs. sincere statement; joy vs. dislike), and is therefore highly clinically significant. Additionally, subtle differences in prosody have also been reported among clinically unaffected first-degree relatives, and could constitute a marker of underlying genetic liability to ASD [14].

Although a number of studies have provided important insights into the characteristics of speech prosody in individuals with ASD, little is known concerning the underlying causes of prosodic differences in ASD. Moreover, most studies have concentrated on studying prosody in English-speaking groups [2, 6]. Language is fundamentally cultural, and how specific aspects of prosody are used can vary substantially across languages [15]. Studying language components, such as prosody, across typologically and prosodically distinct languages is therefore critical to address the variability of ASD symptomatology, and its underlying mechanisms.

Since the earliest delineations of ASD [16], prosodic differences have been described in subjective reports as a characteristic of ASD (see [1] for a review). Subsequent studies applied more objective methodologies examining the physical (acoustic) profile of prosody in ASD, including the fundamental frequency (f0), duration, and periodicity of speech sounds—acoustic properties which are correlates of fundamental aspects of prosody, namely intonation and rhythm. Findings have largely converged, showing differences in intonational properties of prosody (e.g., pitch), in ASD [2, 6]. Evidence suggests that individuals with ASD often demonstrate overall higher f0 (the acoustic correlate of pitch) [17], and larger f0 range at the utterance level [17–21], at the syllabic level [22], at the utterance final position [14], and specifically when using focus to highlight new information [23]. In rhythmic aspects of prosody, speech produced by individuals with ASD also demonstrated less of a distinction in duration between stress and unstressed syllables [22], slower speech rate [14], as well as greater intensity and longer phrase durations [20]. Although these prosodic features do not usually result in unintelligible speech, they are nevertheless perceptible [14] and can contribute to an impression of “oddness” reported by listeners [20, 24, 25].

Traditionally, speech prosody is categorized into affective and linguistic categories [26]. Affective prosody is used to express emotion and communicative intent, both central to social interactions, through non-linguistic elements of speech [27]. Despite some cross-cultural variabilities, fundamental emotions and basic communicative intents expressed through prosody are typically similarly understood by listeners across different linguistic and cultural backgrounds [28, 29], and may even have evolutionary homologies in other mammals [30–32]. In contrast, linguistic prosody is used to represent lexical and grammatical elements of language, and may vary drastically across languages [15, 33]. In particular, while pitch signifies sentence intonation (e.g., to convey a statement vs. a question) in English, pitch also conveys *lexical* meaning in tone languages such as Cantonese. For example, in Cantonese, a syllable /ji/ means ‘to cure’ when produced with a high-level pitch pattern but means ‘two’ when produced with a low-level pitch pattern.

Prosodic expression in individuals with ASD speaking a tone language has been scarcely investigated. One study examined the f0 and duration of the five Thai lexical tones produced by native speakers with ASD [34]. A separate study of Cantonese-speaking individuals with ASD examined f0 measures of sentence final particles (SFPs), which are crucial linguistic markers for conveying pragmatic information in the language [35]. Another study examined lexical tone imitation of Cantonese- vs. Mandarin-speaking children with ASD [36]. These studies revealed findings similar to those reported in English-speaking individuals with ASD, such that the individuals with ASD exhibited higher f0 [34] and larger standard deviation (i.e., larger variability) of f0 [35, 36]. Some language-specific results were also observed, including shorter lexical tone duration in the ASD group [34]. A positive correlation between the number of SFP types produced by individuals with ASD and the standard deviation of F0 was also found [35], suggesting the more diverse the SFP type an individual produced, the more pitch variation could be realized at the utterance-final position which does not alter core sentential content. Some language-specific findings were not consistent with those reported in studies of English. Specifically, while larger f0 range has been repeatedly reported in English-speaking ASD groups [17–19, 21, 22], Thongseitratch and colleagues [34] found a lower f0 range in the Thai-speaking ASD group. A lower f0 range associated with ASD was also found in a study of Japanese speakers [3]. These mixed results from languages other than English suggest that while certain acoustic characteristics of speech may be impacted across languages, typological differences across languages may also result in important language-specific prosodic characteristics associated with ASD. Such differences are not only essential in informing the understanding of prosodic differences as a core feature of ASD, but may also help to optimize culturally sensitive diagnostic and intervention practices.

Taking a cross-linguistic approach, the present study compared prosodic characteristics of speech from individuals with and without ASD across English and Cantonese, two typologically and prosodically distinct languages. English is an Indo-European Germanic non-tone language that belongs to the rhythmic group of stress-timed languages [33], whereas Cantonese is a Sino-Tibetan Sinitic tone language which is syllable-timed [37]. In the general population, even in languages with such distinct properties, considerable cross-linguistic commonalities have been demonstrated in prosody [28, 29, 38–40], particularly in the affective aspect of prosody that is most reflective of differences in ASD (e.g., emotion and intention expression and recognition). Important cross-linguistic variability has also been observed, especially in linguistic prosody [15, 33]. Indeed, prior cross-linguistic comparisons between prosodic characteristics of ASD have been reported in languages which are typologically and prosodically related (e.g., English and Danish, both Germanic languages [41], and Cantonese and Mandarin, both Sinitic languages [36]). However, studying prosody in ASD across languages with more distinct properties, such as English and Cantonese, may help to further reveal core prosodic differences that are robustly expressed across languages, as well as those that are differentially impacted, suggesting phenotypic malleability that could be important to consider in speech and language interventions.

We implemented a series of novel machine learning (ML)-based analytics to examine prosodic characteristics of ASD both within and across the two languages. ML capitalizes on the multivariate nature of acoustics features (e.g., series of features that vary in time or frequency domains rather than single summed or averaged values) that richly represents the dynamicity of speech prosody, and have been proven to be able to delineate the acoustic characteristics of the prosodic profile of ASD [6, 42–44]. We focused on two classes of acoustic features, namely those relevant to 1) *rhythm*, and 2) *intonation*, considered to be the two core elements of speech prosody [12]. A series of supervised ML models were trained to make classifications of diagnosis (ASD vs. typical development [TD]) using a series of multivariate acoustic features associated with intonation and rhythm, derived from utterance samples of a structured narrative task elicited separately in English and Cantonese. [45–47]. ML classification models have been used successfully to classify individuals with ASD from those with TD using a variety of social-behavioral and neurocognitive measures (see [48] for a review). ML classification models have also proven to be effective in classifying diagnostic status of various psychiatric, cognitive, and speech-language impairments using speech acoustic features [49–51]. In particular, a recent ML study successfully classified ASD/TD using f0-based acoustic features derived from recordings of individual words elicited in a picture-naming task [44]. Capitalizing on the power of the ML classification approach, our ML models examined which class(es) of acoustic features (*rhythm* and/or *intonation*) derived from larger temporal windows (i.e., from utterances elicited in a more dynamic language narrative task) could reliably classify individuals with ASD vs. individuals with TD, both within and across English and Cantonese.

## Materials and methods

### Participants

This study included participants from two languages groups, consisting of native speakers of American English (henceforth, English group) and Hong Kong Cantonese (henceforth, Cantonese group). The English group included 55 individuals with ASD (English ASD group) and 39 individuals with TD (English TD group). The Cantonese group included 28 individuals with ASD (Cantonese ASD group) and 24 individuals with TD (Cantonese TD group).

We acknowledge the variability in preferences related to person-first and identity-first terminology (i.e., individuals with ASD versus autistic individuals). We do not endorse one style over the other. For the purpose of this manuscript, to ensure that language is parallel when referencing both participant groups, we remain consistent with the following terminology: individuals with autism spectrum disorder (ASD) and individuals with typical development (TD).

Participants from the English group were recruited in the United States through a larger family-genetic study of ASD, which included individuals with ASD, their parents, and respective controls. Participants from the Cantonese group were recruited in Hong Kong using advertisements posted on social media platforms (e.g., Facebook) and directly sent to schools and organizations with existing populations of individuals with ASD, as well as from employment programs particularly designed for adults previously diagnosed with Asperger syndrome or high-functioning Autism. Informed assent/consent was obtained from all participants and guardians (as applicable), and procedures were approved by the respective institution’s ethics committee. All procedures were in accordance with ethical standards of the institutional and/or national research committee and with the 1964 Helsinki declaration and its later amendments or comparable ethical standards.

Only participants having no reported history of brain injury, major psychiatric disorder, known genetic syndrome, or neurodevelopmental disorder (other than ASD) were included. Participants with TD in both language groups were screened for personal or family history of ASD or related genetic disorders (e.g., fragile X syndrome). ASD status was confirmed with research reliable administration and scoring of the Autism Diagnostic Observation Schedule 2nd Edition (ADOS-2). ADOS-2 Overall, Social Affect, and Restricted and Repetitive Behaviors (RRB) calibrated severity scores were used to determine ASD severity [52].

To assess IQ, the Wechsler Abbreviated Scale of Intelligence (WASI; Wechsler 1999), Wechsler Adult Intelligence Scale-Third or Fourth Editions (WAIS; Wechsler 1997, 2008), or the Wechsler Intelligence Scale for Children-Fourth Edition (WISC-IV; Wechsler 2003) were administered to participants in the English group. The Test of Nonverbal Intelligence, Fourth Edition (TONI-4) was administered to participants in the Cantonese group.

### Narrative elicitation

Participants were asked to narrate (in their respective languages) the 24-page wordless picture book, *Frog, Where Are You?* [53]. The book presents a story about a boy and his dog, who are searching for the boy’s missing pet frog. This book has been used extensively in studies of narrative discourse in ASD and other neurodevelopmental disabilities [14, 54–58] and in cross-linguistic work [59]. Participants were asked to narrate the story as each page of the book was presented to participants on a computer monitor, while their narrations were audio recorded. No examples were provided to the participant. The recordings were first transcribed and segmented into individual utterances, defined by natural pauses. Twenty utterances from participants (English ASD n = 33; English TD n = 33; Cantonese ASD n = 24; Cantonese TD n = 24, Table 1) were selected for subsequent analyses (see §1.1 in S1 File for further description of acoustic data collection and processing).

**Table 1:** Demographic information.

	ASD (Cantonese)	TD (Cantonese)	ASD (English)	TD (English)
	M (S.D.) Range	M (S.D.) Range	M (S.D.) Range	M (S.D.) Range
Males: females (Count)	19:9	17:7	29:4	15:18
Chronological age	17.83 (9.24) 8–32	18.88 (8.70) 8–31	**15.96 (7.38)** 6–35	**19.31 (5.35)** 12–32
IQ	**108.54 (10.92)** 84–127	**115.08 (10.13)** 92–128	*104.13 (14.34)* 73–131	*111.03 (13.79)* 79–143
ADOS-2 Total Severity Score	6.00 (2.33) 3–10		7.02 (1.85) 3–9.5	

**Bold**: Significant differences as per t-tests (*p* < 0.05) between the ASD and TD groups within the respective language group. *Italics*: Marginal differences as per t-tests (0.05 > *p* < 0.1) between the ASD and TD groups within the respective language group. ASD: Autism Spectrum Disorder, M: Mean, S.D.: standard deviation, TD: typical development

The narrative data elicited from the English group were the focus of a prior published study [14], examining syllable-level acoustic measures that were not included in the present analyses. The English participant narrative speech samples were included in this study for cross-linguistic comparison only focused on utterance-level acoustic measures that have not been previously studied. In contrast to recent studies in both English [14] and Cantonese [35] reporting prosodic differences associated with ASD at language-specific syllabic-level units (final-syllable excursion for English; SFP for Cantonese), the current study focused on the *utterance* level. In contrast to syllabic-level units, the *utterance* is associated with the intonational phrase, the most fundamental unit of prosody argued to be universal across all languages [60–63]. The ML approach allowed us to perform classifications using multivariate acoustic features representing the prosodic dynamics over the entire duration of utterances (described in the following section), therefore enabling us to examine, at the most fundamental prosodic unit common among English and Cantonese, the extent to which patterns of speech prosodic differences in ASD similarly manifest across different languages.

### Acoustic feature extraction

Two classes of multivariate acoustic features were extracted from utterances in the narrative samples utilizing the MATLAB Audio Toolbox and MATLAB scripts provided by prior studies [45–47, 64].

1. Speech Rhythm: Speech rhythm is traditionally considered as durational variations across syllables in an utterance that signal linguistic and affective properties [12]. The temporal envelope of speech signals, in general, represents the evolution of the speech signal waveform amplitude over time that displays temporal regularities correlating to the syllabic rhythm of the signal [64]. Such rhythmicity, especially those of 2–8 Hz, is crucial in the neural processing of intelligible speech as it aligns with in brain areas in the spoken language processing pathway that oscillate at a similar rate [65]. Three measures, namely 1) envelop spectrum (ENV), 2) intrinsic mode functions (IMF), and 3) temporal modulation spectrum (TMS) [45–47] were derived from all utterances of each participant to comprehensively capture aspects of speech rhythm represented in the temporal envelope. An overall of 8640 these rhythm-relevant features were extracted across the 20 utterances from each participant.
2. Intonation: Speech intonation, by definition [61], refers to the variation of voice pitch across time. The major acoustic correlate of pitch is the fundamental frequency (f0), which is a simple yet robust index of speech intonation that has repeatedly been implicated in the prosodic characteristics of ASD [17–21, 44]. In contrast to univariate measures (single values) of f0 used in prior studies [17–21], often computed by taking the mean or variance over multiple temporal windows or across speech samples, we focused on entire f0 contours, i.e., a series of time varying f0 values (i.e., multivariate) across the entire duration of each individual utterance. The f0 contour represents essential elements of both linguistic prosody and affective prosody at the sentence/utterance level [11, 61, 66]. In particular, the f0 contour has been the target of study in both empirical and theoretical work of intonational phonology [61, 63], as it encompasses the dynamicity of speech intonation which varies across time within the most fundamental utterance-sized linguistic unit known as the intonational phrase. Therefore, intonation-relevant features examined in this study were comprised of time-varying f0 values derived from narrative utterances. From each utterance, 20 f0 values from a time-normalized f0 contour were extracted and further concatenated across all 20 utterances of a participant, resulting in 400 features for each participant.
  Further technical descriptions of the acoustic feature extraction procedures are presented in §1.2 in S1 File.

### Machine learning classification

In two sets of ML models, we trained a series of linear support vector machine (SVM) classifiers to classify participants with ASD from those with TD using principal components derived from the multivariate acoustic features extracted from participants’ narrative samples. The SVM performed classifications by finding a hyperplane from a multidimensional space that divided data points from the principal components according to participants’ diagnosis.

The SVM was chosen because it is well suited to handle data of high-dimension, and the types of speech acoustic features implicated in ASD (e.g., f0-based features) in particular [44]. The linear kernel was chosen due to its effectiveness in handling datasets with a small sample size [67]. Widely chosen in studies with datasets where the number of features often even exceeds that of samples (e.g., neuroimaging [68] and gene expression studies [69]), the linear SVM is preferable to non-linear kernels because theoretically it is always possible to find a linear decision boundary that separates data, in spite of high data dimensionality and small sample size [68].

Several procedures were performed in the classification pipeline to further avoid overfitting and optimistic bias due to limited sample size and the high-dimensionality of data [70] (see §1.3 in S1 File for technical details of procedures in the machine learning classification pipeline). Classifications were performed using a repeated 10-fold cross-validation procedure, in which data reduction (into principal components) and hyperparameter tuning were performed in a nested fashion. Classification performance was quantified as the Area Under the Curve (AUC) of a receiver operating characteristics (ROC) curve computed based on the probability vector of the predicted labels across all cross-validation folds. The accuracy, sensitivity, and specificity values of the classification were also recorded. A permutation approach was used to estimate the statistical significance of each series of classification with the AUC values.

In Model 1, classifications were performed separately on English and Cantonese samples. The comparison between classification performance patterns across the English and Cantonese classifications allowed us to both identify the specific acoustic characteristics associated with prosody in ASD within these two languages, as well as determine whether such characteristics were consistent or different between the two languages. In Model 2, ASD vs. TD classifications were performed on an English and Cantonese-combined dataset in order to identify aspects of prosody that were present in ASD narrative samples across the two languages. In each model, two sets of SVM classifications were performed using the principal components derived from *rhythm*- and *intonation*-relevant features respectively.

## Results

**Fig 1:**
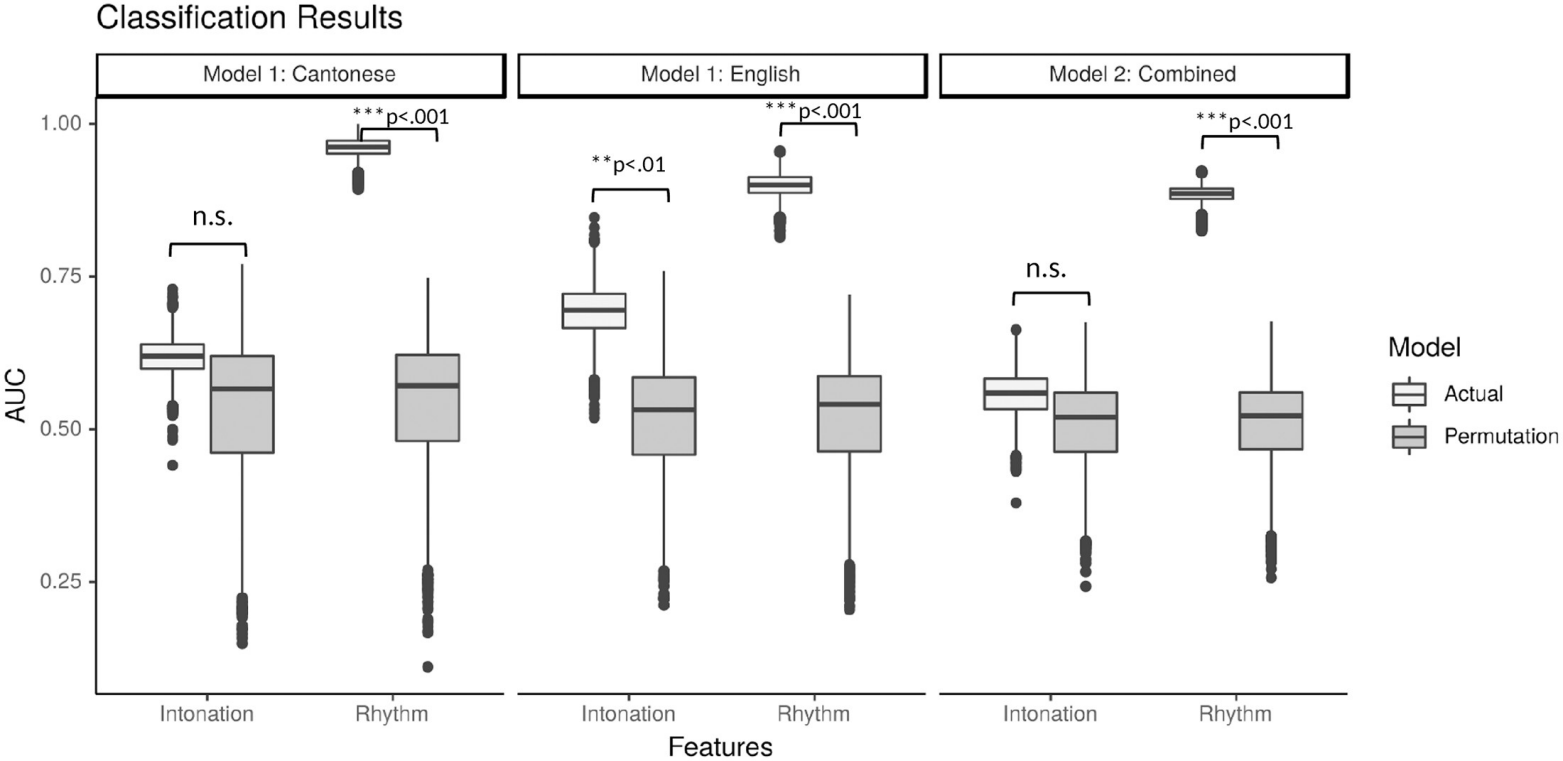
Machine learning classification results. Machine learning classification results displayed in boxplots of Area-Under-the-Curve values across 5001 iterations and permutations.

Model 1 performed classifications between ASD and TD diagnosis using *rhythm*- (ENV, IMF, and TMS) or *intonation*-relevant (f0 contours) features, on English and Cantonese samples *respectively*. Model statistics of all classifications are presented in Table 2. The AUC values of all SVM classifications in Model 1 are presented in Fig 1 (left and middle). The classifications with *rhythm*-relevant features were significant for both English (median AUC = 0.900, *p* < 0.001—the median AUC corresponded to an accuracy [ACC] of 0.819, sensitivity [SENS] of 0.788, and specificity [SPEC] of 0.849), and Cantonese (median AUC = 0.962, *p* < 0.001; ACC = 0.880, SENS = 0.917, SPEC = 0.833). In contrast, classifications with *intonation*-relevant features were significant only for English (median AUC = 0.695, *p* = 0.007; ACC = 0.683, SENS = 0.758, SPEC = 0.606) but not Cantonese (median AUC = 0.620, *p* = 0.507; ACC = 0.605, SENS = 0.667, SPEC = 0.542). A post-hoc analysis (presented in §2 in S1 File) was further performed to rule out gender and age as potential confounding factors in the f0-based classification in English, given differences in gender and age observed between the English (but not Cantonese) groups.

**Table 2:** Model statistics.

Model	Language	Features	Median AUC	ACC	SENS	SPEC
1	English	Rhythm	0.900***	0.819	0.788	0.849
		Intonation	0.695**	0.683	0.758	0.606
	Cantonese	Rhythm	0.962***	0.880	0.917	0.833
		Intonation	0.620	0.605	0.667	0.542
2	English & Cantonese	Rhythm	0.886***	0.835	0.790	0.877
		Intonation	0.559	0.566	0.632	0.509

Model median area-under-the-curve (AUC) and associated accuracy (ACC), sensitivity (SENS), and specificity (SPEC)

***Permutation *p* <.001.

Model 2 further examined the classifications between ASD and TD diagnosis using *rhythm*- or *intonation*-relevant features, using a dataset *combining* both English and Cantonese samples. The AUC values of all SVM classifications in Model 2 are presented in Fig 1 (right). The classifications using *rhythm*-relevant features was significant (median AUC = 0.886, *p* < 0.001; ACC = 0.835, SENS = 0.790, SPEC = 0.877), whereas the classification using *intonation*-relevant features was near chance level (median AUC = 0.559, *p* = 0.509; ACC = 0.566, SENS = 0.632, SPEC = 0.509).

**Fig 2:**
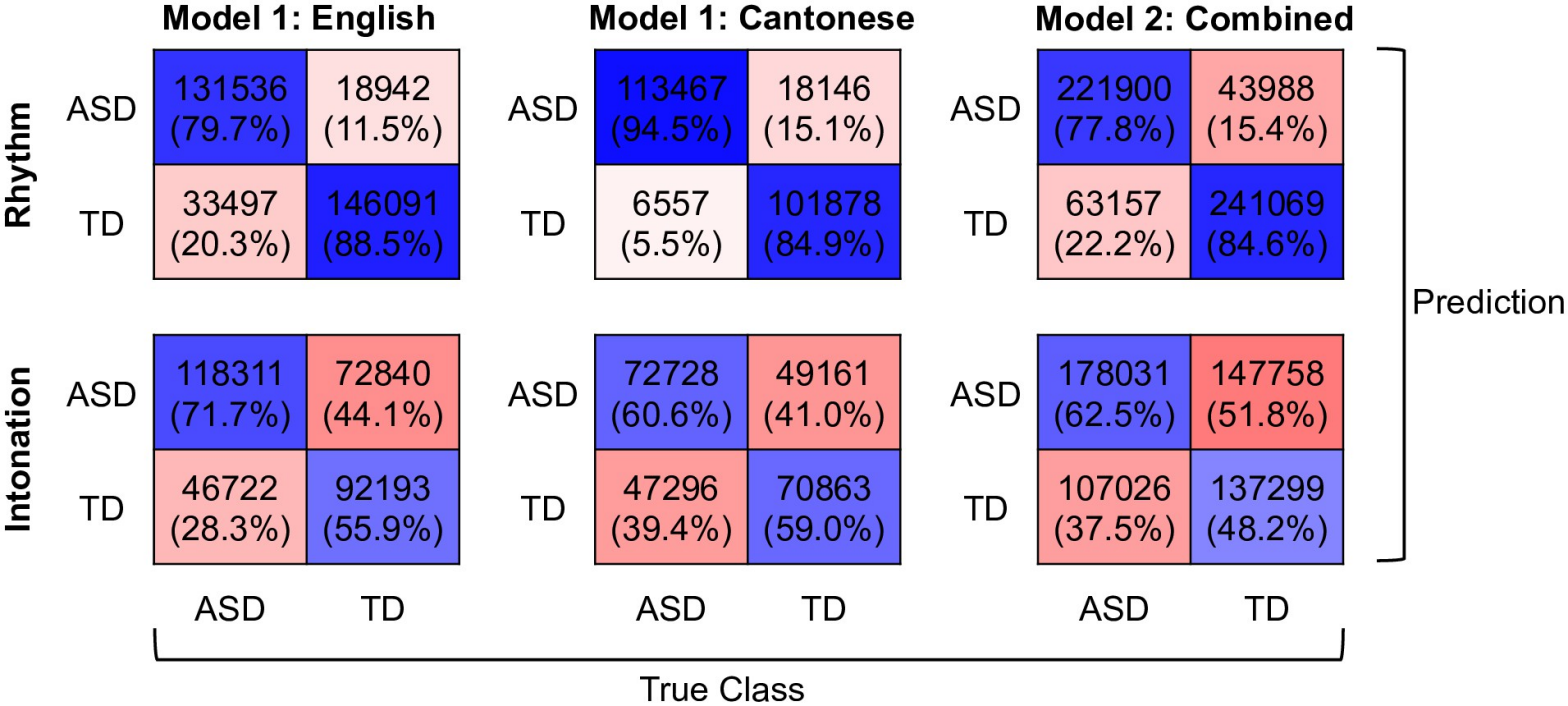
Confusion matrices. Confusion Matrices of machine learning classifications in Model 1 (English and Cantonese) and Model 2 (Combined), aggregated across all 5001 iterations of cross-validation in each classification. Blue hues: correct predictions; Red hues: incorrect predictions.

Confusion matrices of all classifications are presented in Fig 2. In general, all classifications using rhythmic features showed comparably high SENS (accuracy in predicting ASD cases) and SPEC (accuracy in predicting TD cases) rate. SPEC is higher in Model 1 on English and Model 2, whereas SENS was higher for Model 1 on Cantonese. In contrast, although SPEC was low in the statistically significant Model 1 on English using intonational features, good SENS was observed. The non-significant classifications using intonational features had generally low SENS and SPEC.

## Discussion

The present study applied machine learning (ML)-based analyses to acoustic features of speech in ASD, in an attempt to identify prosodic differences in ASD that span two typologically distinct languages, as well as those that may be shaped by specific linguistic and cultural influences. Capitalizing on the power of ML algorithms for performing classifications using high dimensional data [6, 42–44], we examined acoustic features representing intonational and rhythmic aspects of prosody across time or frequency domains. Findings from ML-based algorithms demonstrated that acoustic features can be used to reliably classify ASD vs. TD group status in both English and Cantonese. Results demonstrate the value of moving beyond more reductionist approaches (e.g., averaging or picking extreme values) often used in traditional univariate acoustic analyses on pitch, stress, and speech rate [2], to capture the dynamicity of speech prosodic profiles, and point to differences in prosodic features that may be robust characteristics of the ASD speech and language phenotype in multiple languages.

Specifically, results of Model 1 indicated that prosodic features in the rhythm of speech(i.e., the envelop spectrum [ENV], intrinsic mode functions [IMF], and temporal modulation spectrum [TMS]—representations of temporal regularities of speech signal waveforms correlating to the syllabic rhythm) contained crucial information captured by the ML algorithm to differentiate individuals with ASD from controls. Such findings complement the many rich, descriptive studies of prosody conducted previously, that described differences in stress pattern, speech rate, and loudness that could be attributed to ASD [4, 71–75]. It is notable that the ML algorithms identified in this study appeared to capture patterns from acoustic features that were representative of these prosodic characteristics, paralleling such descriptive observations by clinicians and researchers. Results of Model 1 also converge with using objective acoustic measures to characterize speech rhythm in ASD, which have strongly implicated that acoustic measures of speech rate and lexical stress based on stressed syllable duration, f0, as well as cross-syllabic durational variability (e.g., the Normalized Pairwise Variability Index) varied as a function of ASD diagnosis [14, 22].

Perhaps a more revealing aspect of Model 1 is its cross-linguistic design, examining both English and Cantonese utterance samples. Relatively few studies [3, 34–36] have examined prosody in ASD in languages prosodically and typologically distinct from English [41]. All of these studies focused only on pitch, and only one [36] applied a cross-linguistic design to allow direct comparisons of prosody across multiple languages, in the same speech sampling context. The ML algorithm employed in Model 1 was able to classify ASD diagnosis using rhythm-relevant acoustic features (ENV, IMF, and TMS) derived from both English and Cantonese, revealing strong performance for classification (median AUCs ∼ 0.9) in both languages respectively. Given the considerable typological differences between English and Cantonese, it is notable that the same type of acoustic features derived from English and Cantonese produced reliable ASD/TD classifications, suggesting that speech rhythm is an important feature of the prosodic profile of ASD that is evident in multiple languages. In Model 2, with datasets combined, the classification only using the rhythm-relevant acoustic features was also robust (median AUC > 0.8), particularly considering the large linguistic and acoustic variability introduced to the dataset by collapsing the two languages (see §3 in S1 File for a supplementary ML analysis demonstration of systematic cross-linguistic differences present in our rhythm-relevant features). These results suggest that there are rhythmic characteristics of prosody associated with ASD in both English and Cantonese, that potentially represent aspects of a meaningful prosodic profile in ASD that is robustly expressed across the two typologically distinct languages. These results also complement prior cross-linguistic work reporting common rhythmic-related characteristics (i.e., pause length) across languages, i.e., English and Danish [41]. Identification of such a constellation of acoustic features that can reliably predict ASD diagnostic status, across different languages, may hold significant potential for contributing to diagnostic and intervention practices, as well as studies to understand the basis of potential language-related impairments in ASD.

Such cross-linguistic commonalities in rhythmic characteristics of prosody associated with ASD may partly stem from the crucial role of prosody in social communication that is partly invariant across cultures. Speech prosody, in general, demonstrates strong similarities across cultures and languages in terms of the ability to recognize emotion from prosody [28]. Such invariance may even be contributed by the biological depth of prosody in potentially having evolutionary homologies among primates [30], as demonstrated by comparative studies showing parallels in the ways in which humans and animals shape spectro-temporal features of their vocalizations to express emotion [30–32]. To further understand the biological significance of prosodic differences (specifically with regard to rhythm) in ASD, future studies should examine the structural and functional neurophysiology associated with the production of prosody in speakers of multiple languages. For instance, functional neuroimaging studies have found increased right inferior frontal gyrus [76] and right caudate activation in individuals with ASD when performing prosody comprehension tasks [77], which were interpreted as evidence of more effortful processing needed to interpret prosodic cues. Such findings would be particularly compelling if replicated in individuals with ASD who are speakers of languages other than English.

In contrast with strong performance for classifications using rhythm-relevant acoustic features in both languages in Model 1, classifications using intonation-relevant acoustic features (timing-varying f0 values) were significant for English, but not Cantonese. Unlike non-tone languages where pitch serves linguistic and para-linguistic functions at word, phrase, and utterance levels, in tone languages such as Cantonese, pitch is also used to convey meaning at the syllabic and lexical levels. Therefore, differences in intonation may manifest differently across languages due to cross-linguistic variability (which is represented in our intonation-relevant features, as suggested by the supplementary analysis presented in §3 in S1 File), into f0 contour patterns that were identifiable by the ML algorithm for English but not for Cantonese. Contrasting prior findings showing common cross-linguistic pitch characteristics in ASD among typologically and prosodically related languages (i.e., English and Danish [41], Cantonese and Mandarin [36]), the cross-linguistic differences between English and Cantonese may have contributed to the lack of significant classifications using intonation-relevant features in Model 2, where no common feature patterns consistently associated with ASD across the two languages combined could be identified.

One consideration in cross-linguistic variability observed in ASD intonation is whether intonation differences in ASD are apparent only in speakers of English (or its typologically related languages [41]). Indeed, previous acoustic analyses only reported utterance-level f0 differences across ASD and TD groups in English [17–19, 21, 22] but not Cantonese [35], consistent with our classification patterns using f0-based features across Models 1 and 2. It is possible that the prolific usage of linguistic pitch in tone languages provides a compensatory effect ameliorating intonational differences in ASD. In the perception domain, pitch processing differences found in tone language-speaking children with ASD [78–81] were surprisingly not evident in their adult peers [82], potentially due to a longer exposure to the native tone language. This possibility, i.e., that extensive pitch experience may ameliorate intonational differences in ASD, highlights pitch and intonation as a fruitful target for speech interventions in ASD for those who do not speak a tone language, where targeting this potentially more malleable factor could lead to therapeutic gains and help to advance more global speech and language characteristics. From a genetic perspective, in contrast to people of European descent, most Han Chinese people are carriers of the T allele of the ASPM gene that favors the ability to process linguistic pitch patterns [83, 84]. This may implicate a role of genetic factors in contributing to cross-linguistic differences of ASD phenotypes, such as by potentially ameliorating intonational differences in our participants from our Cantonese ASD group, all of whom reported to be of Han Chinese descent. Future studies might examine the *ASPM* gene as a potential genetic marker for prosodic differences in ASD, shedding light onto the etiologies of communications disorders [85] that contribute to distinct cross-linguistic patterns of intonation in ASD.

### Limitations

Several potential limitations should be considered in interpreting findings. First, there was a wide age range across our Cantonese and English groups and a small number of females included in the current study. With exhaustive efforts made to avoid model overfitting (i.e., the choice of linear SVM, repeated cross-validation, data reduction, and hyperparameter tuning in a nested fashion), robust classification results were found, potentially representing aspects of prosodic profiles of ASD which are relatively age and gender invariant. Moreover, neither age nor gender appeared to influence classification results, as per our post-hoc analysis (§2 in S1 File). Nevertheless, future studies with larger samples are needed to confirm whether findings reported here extend to both males and females and different age ranges where prosodic abilities may vary. Relatedly, future studies should employ more comprehensive assessments of language abilities for group matching, since baseline language ability may covary with age and autism severity [86, 87], influencing prosody and pitch in particular [88]. Finally, it will be important for future work to examine languages from typological classes beyond English and Cantonese. Such work will optimally include much larger, well matched corpora of multi-linguistic narrative samples from individuals with ASD, with potential to shed crucial insight into whether the rhythmic commonality identified here may represent features of ASD that are expressed across languages, despite cross-linguistic variations in prosodic properties.

## Conclusion

Using brief utterance samples from a structured narrative task, ML models applied in this study were able to robustly classify individuals with ASD from those with TD (reflected by median AUC values ∼0.9), based on rhythm-relevant features in both English- and Cantonese-speaking populations. The success of our ML approach yields implications for its future clinical utility, such as developing automatic detection of ASD to augment diagnosis [44] for personalizing therapy and training regimens [89]. Although further study with larger samples, and with additional language comparisons, is needed to support adaptation of the current models into clinical use, the current findings highlight the potential of ML-based methods to objectively identify other speech language-relevant differences in ASD (and potentially other neurodevelopmental disabilities impacting speech and language) using acoustic data [49]. Further, speech samples were relatively convenient and efficient to obtain, rendering these methods relatively feasible and potentially high yield for clinical application, and highlighting the promise of optimizing ML-based diagnostic models that make use of speech acoustic data as clinical tools. Future studies might also apply ML-based approaches to stratify populations for target in intervention and biological studies alike.

Together, findings from this study provide some of the first evidence for cross-linguistic commonalities in rhythmic characteristics in ASD across two typologically and prosodically distinct languages, while suggesting intonational characteristics in ASD are different across the two languages. Future studies examining languages rhythmically similar to English (e.g., German and Dutch), as well as those distant from both English and Cantonese (e.g., Japanese), could provide additional crucial insights into establishing prosodic profiles of ASD (e.g., rhythm) which could potentially be invariant across languages, while highlighting those (e.g., intonation) potentially more malleable to be shaped by language-specific linguistic properties.

## Supporting information

S1 File Supplementary methods, post-hoc analysis, and supplementary machine learning analysis. (PDF)
