# Supplementary material for: Cross-linguistic patterns of speech prosodic differences in autism: A machine learning study
Source: PLoS One. 2022 Jun 8;17(6):e0269637. doi: 10.1371/journal.pone.0269637 (PMC9176813; doi:10.1371/journal.pone.0269637)
Supplement: S1 File — (PDF) [file pone.0269637.s001.pdf]

Supplementary Information

1 Supplementary Methods:

1.1 Narrative utterance pre-processing and selection

Narrations were recorded in stereo using a table microphone positioned approximately 8 inches from the participant's mouth during the narration and digitized with a sampling rate of 48,000 Hz using a personal computer. Recordings were then pre-processed using the program Praat [1]. Stereo recordings were first converted to mono for subsequent analyses by averaging across the two channels. The recordings were transcribed verbatim from video by transcribers blind to group status using Systematic Analysis of Language Transcripts (SALT) or ELAN (English) or CLAN (Computerized Language ANalysis). Thirteen percent of First telling files were randomly selected to assess word-word agreement, and mean reliability across all files was 95% (range = 90%-99%). Then, utterances (defined by natural pauses) of the recordings that correspond to the narratives were manually segmented using Praat Textgrids. For each participant, only the first utterance from each page of *Frog, Where Are You?* was extracted. Utterances containing character speech, a question, unfinished words, an interruption by the examiner, fewer than two words, shorter than 1 second, unintelligible or directed towards someone else in the room and not related to the narrative, or abandoned, were excluded [2]. This resulted in a maximum of 24 utterances from each narration, each with a structure that typically corresponded to a maximal intonational phrase (i.e., the highest unit of structure within linguistic models of the prosodic hierarchy [3]).

To ensure comparability across participants, both across language (English vs. Cantonese) and diagnosis (ASD vs. TD), only the first 20 utterances from each participant's narrative were included in the analysis

(mean length: 5.85 sec, S.D. 3.63). Participants without a minimum of 20 qualifying utterances (English ASD n=16, English TD n=6, Cantonese ASD n=4) were excluded, resulting in final sample sizes of: English ASD n=33; English TD n=33; Cantonese ASD n=24; English TD n=24.

1.2 Acoustic feature extraction

For each of the 20 utterance samples from every participant's narrative data, the following series of acoustic features were extracted:

1. *Rhythm:* Three types of acoustic features were derived from all utterances to comprehensively capture aspects of speech rhythm.

   i. The speech envelope spectrum (**ENV**) represents temporal regularities correlating to rhythmic properties of the signal [4, 5]. For each utterance, the vocalic energy amplitude envelope was first derived. To derive the envelope, the raw time series of the utterance was first chunked into consecutive bins of one second. Following Tilsen and Arvaniti [5], the time series of each chunk was filtered with a passband of 400-4000 Hz to de-emphasize non-vocalic energy such as glottal energy (including the f0) and obstruent noise. The bandpass-filtered signal was then low-pass filtered with a cutoff of 10 Hz to represent the envelope. The frequency decomposition of the envelope was then computed. First, the envelope was downsampled by a factor of 100 and windowed using a Tukey window ($r = 0.1$) to aid further spectral analyses. The envelope was then normalized by subtracting the mean and rescaled to have minimum and maximum values of -1 and 1 respectively. A fast Fourier transform was first applied to the normalized envelope which was also zero-padded to a 2048-sample window. The spectra across all one-second chunks of each utterance were then averaged to form the envelope spectrum of the utterance, each consisting of 1660 values.

   ii. The intrinsic mode functions (IMFs) were further computed from the time-varying speech envelope (as described above) using empirical mode decomposition (EMD), representing syllabic and supra-syllabic-level fluctuations relevant to speech rhythm [6]. The frequency decompositions of **IMF1** and **IMF2** (i.e., the averaged power spectrum density of 1-10 Hz from

the frequency decomposition all IMF1s and IMF2s across all 1-second envelop chunks of each utterance) were included as features, each consisting of 1660 values.

iii. The temporal modulation spectrum (**TMS**) is the frequency decomposition of the temporal envelope of a signal that reflects how fast sound intensity fluctuates over time [7]. Temporal modulation of lower frequencies ($<$ 32 Hz) is a primary acoustic correlate of perceived rhythm in speech [8, 9], which contributes to speech intelligibility [10]. For each utterance, the raw time series was first chunked into consecutive bins of 1 second. The TMS of each 1-second bin was then computed using the procedure and MATLAB script from Ding and colleagues [7]. In the procedure, the sound signal in each bin was first decomposed into narrow frequency bands using a cochlear model and then from each band the temporal envelope was extracted. The extracted envelopes were rescaled using a logarithmic function, and were then converted into the frequency domain by the Discrete Fourier Transform (DFT). The TMS was the root-mean-square of the DFT of all narrowband power envelopes. The TMS of each narrative sample was taken as the average TMS of all bins. The TMS of each utterance consisted of 2000 values.

2. *Intonation:* The fundamental frequency (f0) contour for each utterance were derived to represent its intonation. For each utterance, a raw f0 contour was first derived using the `pitch` function of the Audio Toolbox in MATLAB [9]. f0 values of the contour were estimated using a Normalized Correlation Function algorithm [11] with analysis windows that spanned 52 ms and overlapped with adjacent analysis windows for 42 ms. To minimize pitch tracking errors, pitch tracking ranges dependent on speaker age and gender (see Supplementary Table 1) were implemented in the algorithm [2]. Given that the duration of each utterance varied, a time normalization procedure [12, 13] was further performed to obtain an f0 contour that was uniform in size across all utterances. The procedure took 20 f0 values of the raw f0 contour at equal proportional intervals. The 20 f0 values were then concatenated to form a time-normalized f0 contour.

For each type of acoustic measure, the features of all 20 utterances from each participant were concatenated. This resulted in 400 *intonation-relevant* f0 features per participant. The ENV, IMF1, IMF2,

and TMS features were further concatenated in each participant, resulting in 8640 *rhythm*-relevant features per participant.

1.3 Machine Learning Classification Pipeline

All ML procedures were performed in MATLAB, including functions from the *Statistics and Machine Learning Toolbox*. In both Model 1 and Model 2, a total of two set of classifications were performed for each machine-learning model, using features relevant to *intonation* and *rhythm* respectively.

For each set of classifications, a linear SVM classifier was trained to classify ASD vs. TD diagnosis groups based on each participant's acoustic feature input. To objectively evaluate the performance of SVM classification and to minimize optimistic bias and overfitting in the classification [14, 15], a repeated 10-fold nested cross-validation (CV) procedure was performed for 5001 iterations. In each iteration, all samples (the feature sets from all ASD and TD group participants combined) were randomly divided into 10 stratified partitions, using the `cvpartition` function. Then, a linear SVM classifier was iteratively trained using input features from 9 out of 10 partitions (the training set) and the diagnosis labels (ASD and TD). Before the training, a Principal Component Analysis was first performed on the features of the training set to reduce data dimensionality, using the `pca` function and its default parameters. The scores for all highest ranked principal components (PCs) which, in combination, explained 99% of the total variance of the training set were used for further training. Hyper-parameter tuning on the regularization (C) parameter of the linear kernel was performed using a grid search approach (from an array of [.01,.1,1,10,100]) to identify the optimal C parameter which achieved the least classification loss in a nested 10-fold cross-validation within the training set using the PC scores. A linear SVM was then trained using the optimal C parameter on the entire training set of PC scores, using the `fitcsvm` function. This training was validated by generalizing to the remaining one partition (testing set), i.e., whether the trained model accurately predicted the diagnoses of participants in the testing set, using PC scores projected from features in the testing set with the PC coefficients derived during the model training. This training-validation process was repeated for 10 times until features of all 10 folds had been tested against each other. Classification performance was quantified as the Area Under the Curve (AUC) of a receiver operating characteristics curve computed based on the probability vector of the predicted labels across all 10 folds of the cross-validation, while their corresponding classification

accuracy, sensitivity, and specificity values were also recorded. A total of 5001 iterations performed yielded a distribution of 5001 AUC values.

Statistical significance of each set of classifications was assessed using a permutation approach. A null distribution of AUC values was computed by repeating the same cross-validation procedure for 5001 times with the diagnosis labels of participants randomized each time. The percentage of AUC values from the permuted model that was equal to or higher than the median AUC from the actual classification was taken as the p-value [16]. To adjust for multiple comparison of a total of 2 sets of classifications (using features relevant to *intonation* and *rhythm* respectively), each p-value was adjusted using a Bonferroni correction.

2 Post-hoc analysis: ruling out gender and age effects on f0 feature English ML classification

Results of Model 1 suggest that f0 features can be used to effectively classify ASD and TD diagnostic categories in English samples but not in Cantonese samples. Because of uneven gender ratios across groups (see Table 1 of main article), fundamental gender-related differences in pitch level could have been a confounding factor that impacted f0-based ASD vs. TD classifications (although potential gender-related f0 differences did not drive successful classification in Cantonese samples using f0 features, likely due to our resampling procedure of subjects in addition to the smaller sample size of adults where larger gender differences were expected). Further, the significant age differences across English ASD and TD groups (see Table 1 of main article) may have increased the discriminability of pitch across the two groups, as compared to Cantonese ASD and TD groups which did not differ significantly in age. To rule out gender and age as potential confounding factors in the f0-based classification in English, a post-hoc analysis employing a resampling procedure using age-matched males and females was performed.

2.1 Methods: Matching males and females and their age with resampling

In the post-hoc analysis, 5001 iterations of SVM classification using f0 features was performed according to the nested 10-fold CV procedures described in the ML analysis pipeline. Yet, before each iteration of

classification, the feature array of f0 was resampled such that the male-to-female ratios of samples across ASD and TD groups were the same in the array. This resampling process involved randomly selecting samples of 4 out of 18 females with to match those of the 4 females with ASD. Vice versa, samples from 15 out of 29 males with ASD were randomly selected to match with those from the 15 males with TD. The age of all participants from the two resampled groups were then submitted to a two-sample t-test. In the iteration, the random resampling procedure was performed again until the age differences of the resampled ASD and TD groups were not significant, based on a stringent criteria of ($p > .500$) of the t-test. As a result, the feature array in each iteration consists of f0 features from 38 subjects, with both ASD and TD *age-matched* groups each consisting of 15 males and 4 females. The permutation procedure with 5001 iterations were also performed on the resampled feature array in each iteration of resampling. The p-value of the classification was taken as the percentage of AUC values from the permuted model that were equal to or higher than the median AUC of the actual classification.

2.2 Results: Gender- and-age-matched SVM Classification using f0 features

The AUC values of all SVM classifications in this post-hoc analysis are presented in Supplementary Figure 1 (left). With gender- and age-matched samples through resampling, the SVM classification using f0 features was significant ($p = 0.0028$), achieving a median AUC of 0.833 (corresponding to an accuracy of 0.775, sensitivity of 0.650, and sensitivity of 0.944) which was comparable with, if not qualitatively higher than, the AUC of Model 1 in which gender and age was not matched. This result suggests that neither gender nor age was likely to be a confounding factor in the ASD/TD classification using f0 features derived from English narrative samples.

3 Supplementary ML analysis: demonstration of cross-linguistic acoustic variability using a ML approach

We assumed that both intonational and rhythmic properties vary systematically across our English and Cantonese utterance samples. To test our assumption that such systematic variability was represented in our

speech samples of these two languages, a supplementary analysis was performed. In this analysis, an ML model was trained to classify the two languages using the *intonation* and *rhythm* features derived from individuals of English TD and Cantonese TD groups.

3.1 Methods: Matching English and Cantonese individuals with TD with resampling

Two set of classifications were performed for each machine-learning model, using features relevant to *intonation* and *rhythm* respectively.

In each set of classifications, a total of 5001 iterations of SVM classification was performed according to the the nested 10-fold CV procedures described in the ML analysis pipeline, but this time to classify the two languages. Before each iteration, an undersampling procedure was performed on the English TD group to randomly select 24 samples (out of the overall 33), so as to produce a balanced dataset by matching the smaller number of samples from the Cantonese TD group (N=24). Therefore, the feature array in each iteration of SVM classification consist of input features from 48 subjects. The permutation procedure with 5001 iterations was also performed on the resampled feature array in each iteration of resampling. The p-value of the classification was taken as the percentage of AUC values from the permuted model that were equal to or higher than the median AUC of the actual classification.

To adjust for multiple comparison of a total of two sets of classifications (on *intonation* and *rhythm* features respectively), each p-value was adjusted using a Bonferroni correction.

3.2 Results: Robust language classification using intonation- and rhythm-relevant features

The AUC values of all SVM classifications in this supplementary analysis are presented in Supplementary Figure 1 (left). The SVM classifications using *intonation*- and *rhythm*-relevant features were both significant ($ps < 0.001$), achieving median AUCs of 0.879 (corresponding to an accuracy of 0.800, sensitivity of 0.792, and sensitivity of 0.792) and 0.955 (corresponding to an accuracy of 0.870, sensitivity of 0.875, and sensitivity of 0.875), respectively. This robust classification confirmed our assumption that cross-linguistic

variability in both *intonation* and *rhythm* between English and Cantonese is well represented in the acoustic features extracted from our utterance samples across the two languages.

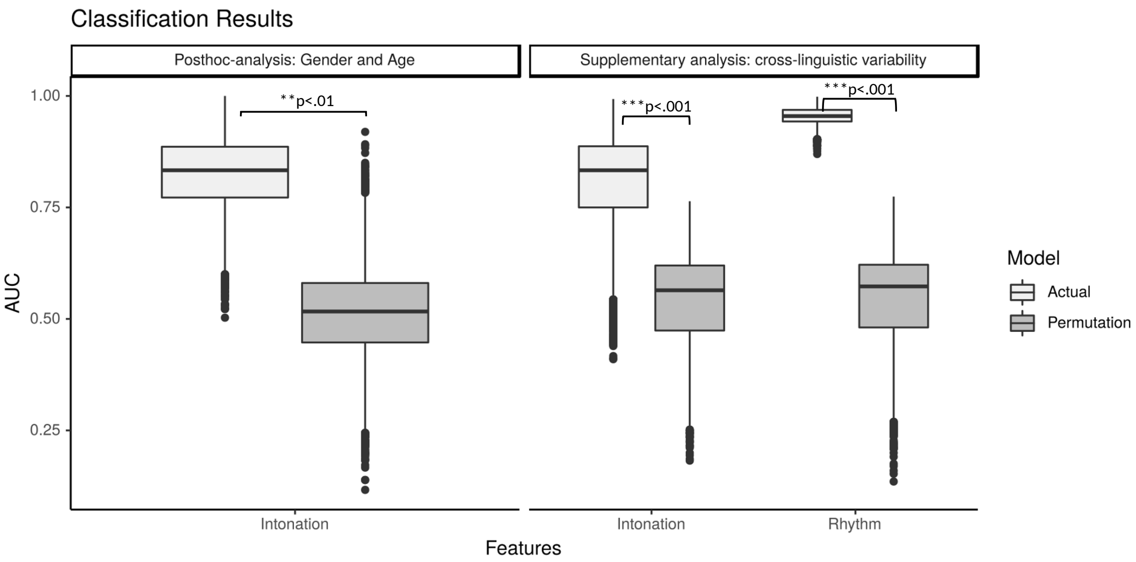

Supplementary Figure 1: Machine learning classification results displayed in boxplots of Area-Under-the-Curve (AUC) values across all 5001 iterations and permutations in each series of classification of the post-hoc analysis controlling for gender and age (left and middle) and supplementary analysis demonstrating cross-linguistic variability (right)

Supplementary Table 1: Fundamental frequency (f0) detection range

	Minimum (Hz)	Maximum (Hz)
Males $<$11 years of age	130	400
Males 12-18 years of age	70	400
Males $>$19 years of age	70	250
Females (all ages)	130	400

References

[1] Boersma, P., Weenink, D.: Praat: doing phonetics by computer [computer program]. Version 5.3.73, retrieved 21 April 2014 from http://www.praat.org/ (2014)

[2] Patel, S.P., Nayar, K., Martin, G.E., Franich, K., Crawford, S., Diehl, J.J., Losh, M.: An acoustic characterization of prosodic differences in autism spectrum disorder and first-degree relatives. Journal of Autism and Developmental Disorders, 1–14 (2020)

[3] Selkirk, E.: 14 the syntax-phonology interface. The handbook of phonological theory, 435 (2011)

[4] Poeppel, D., Assaneo, M.F.: Speech rhythms and their neural foundations. Nature Reviews Neuroscience, 1–13 (2020)

[5] Tilsen, S., Johnson, K.: Low-frequency fourier analysis of speech rhythm. The Journal of the Acoustical Society of America **124**(2), 34–39 (2008)

[6] Tilsen, S., Arvaniti, A.: Speech rhythm analysis with decomposition of the amplitude envelope: characterizing rhythmic patterns within and across languages. The Journal of the Acoustical Society of America **134**(1), 628–639 (2013)

[7] Ding, N., Patel, A.D., Chen, L., Butler, H., Luo, C., Poeppel, D.: Temporal modulations in speech and music. Neuroscience & Biobehavioral Reviews **81**, 181–187 (2017)

[8] Goswami, U., Leong, V.: Speech rhythm and temporal structure: Converging perspectives? In: Linguistic Rhythm and Literacy, pp. 111–132. John Benjamins, Amsterdam; Philadelphia (2016)

[9] Greenberg, S., Carvey, H., Hitchcock, L., Chang, S.: Temporal properties of spontaneous speech—a syllable-centric perspective. Journal of Phonetics **31**(3-4), 465–485 (2003)

[10] Elliott, T.M., Theunissen, F.E.: The modulation transfer function for speech intelligibility. PLoS comput biol **5**(3), 1000302 (2009)

[11] Atal, B.S.: Automatic speaker recognition based on pitch contours. The Journal of the Acoustical Society of America **52**(6B), 1687–1697 (1972)

[12] Xu, Y.: Prosodypro—a tool for large-scale systematic prosody analysis. (2013). Laboratoire Parole et Langage, France

[13] Xu, Y., Xu, C.X.: Phonetic realization of focus in english declarative intonation. Journal of Phonetics **33**(2), 159–197 (2005)

[14] Raschka, S.: Model evaluation, model selection, and algorithm selection in machine learning. arXiv preprint arXiv:1811.12808 (2018)

[15] Vabalas, A., Gowen, E., Poliakoff, E., Casson, A.J.: Machine learning algorithm validation with a limited sample size. PloS one **14**(11), 0224365 (2019)

[16] Xie, Z., Reetzke, R., Chandrasekaran, B.: Machine learning approaches to analyze speech-evoked neurophysiological responses. Journal of Speech, Language, and Hearing Research **62**(3), 587–601 (2019)
